# Evaluation of Postharvest Maturity Indices of Commercial Avocado Varieties Grown at Various Elevations Along Lebanon's Coast

**DOI:** 10.3389/fpls.2022.895964

**Published:** 2022-06-15

**Authors:** Maya Salameh, Diana Nacouzi, Georgette Lahoud, Imad Riachy, Walid El Kayal

**Affiliations:** ^1^Faculty of Agricultural and Food Sciences, American University of Beirut, Beirut, Lebanon; ^2^René Moawad Foundation, Beirut, Lebanon

**Keywords:** avocado, ripening, postharvest, maturity indices, geographical locations

## Abstract

Avocado is known to be a climacteric fruit that must be harvested during the suitable physiological maturity stage to achieve the best edible characteristics and reach the required export standards. It is very hard to visually determine the optimum maturity phases in the different avocado varieties for harvesting, especially because of the limited changes in the external fruit morphology during the maturity phase and because the harvest season is extended throughout several months. Therefore, some laboratory analyses are very crucial to determine the best timing to harvest the fruit. The aim of this study was to evaluate the postharvest maturity indices over 3 harvest stages, mainly dry matter (DM), oil content (OC), fruit firmness, titratable acidity (TA), total soluble solid (TSS/Brix), and fruit weight in commercial avocado varieties in regard to different altitudes and agricultural practices. The varieties in this study were as follows: Hass, Lambhass, Ettinger, Fuerte, Pinkerton, Reed, and Horshim growing at different altitudes that ranged from 50 to 400 m in 7 different regions in Lebanon. Statistical comparison of maturity indices under different locations by variety and harvest stage was performed using one-way ANOVA as well as by principal component analysis (PCA). The results showed a high linear correlation between DM and OC over the different harvest stages. During the late harvest stage, the weight showed a negative correlation between fruit firmness and TSS. The minimum oil content % and dry matter % were recorded for Reed variety (8.2 DM and 9.7 OC%) and the highest oil content % and dry matter % were recorded for Fuerte variety (28.5 DM and 21.6 OC%). The data obtained during this study are used to achieve the best edible characteristics and export standards of commercial avocado varieties growing along the Lebanese coast.

## Introduction

Avocado (*Persea americana* Mill.) is a climacteric subtropical fruit that originated in South America that needs a mild climate to grow (Morton, 1987). This is the case with Lebanon's coastal areas that are mainly planted with citrus and banana fruits. According to the ministry of agriculture, the avocado cultivated lands in Lebanon are still small with about 1,200 growers and almost 50% of them are habitats or growing many different crops along with avocado.

The Lebanese avocado industry is currently expanding with an estimated production of 7,000 tons of fruit from an estimated planted area of 1,200 hectares. FAO (2017) reported that global production of avocado was estimated to be around 5.5 million tons in 2016. Due to ongoing investments in this sector, production is expected to exceed 10,000 tons in the next few years. The majority of this production is consumed locally, with only 20% exported, primarily to Gulf countries. Also, as avocado started to gain attention since 2012 in Lebanon, farmers used to follow traditional agricultural practices, and thus, there is a need to implement good agricultural practices (GAPs) to explore export markets. In order to this industrial sector to develop export markets or exploit premium prices in domestic markets by extending the supply season, more stringent maturity standards should be implemented because this cannot be morphologically inspected (Ozdemir and Topuz, 2004).

The time at which the fruit is harvested is related to the processes taking place in the fruit while it is still attached. These include the accumulation of dry matter and fatty acids, increase in sugar content, and decrease in organic acids, and formation of volatile substances (Xi et al., 2016). An increase in Brix degrees is related to the conversion of polysaccharides and organic acids into sugars or short-chained acids (Taiti et al., 2015). Also, acids and carbohydrates are used to provide the energy that the fruit requires during the ripening process (Taiti et al., 2015). These maturity indices differ among avocado varieties where some kinds ripen before the others. For example, Lamb Hass fruits are ready to be eaten firmer than Hass (Dixon et al., 2009).

With exporting being the target of avocado cultivation, specific maturity index levels are required by the external markets focusing on the dry matter and oil content. Other parameters are also measured such as firmness, fruit weight, size, total soluble solid, and acidity (mainly tartaric acid), but DM and OC are considered as the key indicators for avocado harvesting stage.

It has been reported by Lee (1981) that OC is a key maturity index for avocado. The oil content in avocados depends on many factors, such as the agro-ecological conditions and the fruit development stage. Minimum and maximum oil content percentages for each variety have been established as international standards ranging between 8 and 20% (Ozdemir and Topuz, 2004). However, it is time-consuming and requires the use of toxic solvent and relatively expensive methodology. Therefore, the most popular maturity benchmark for avocado producers around the world is dry matter, which has virtually supplanted oil content. Dry matter is thought to be faster, cheaper, safer (no toxic solvents necessary), lower-tech, and better suited to growers or packhouses for maturity monitoring than oil content (Lee et al., 1983).

Minimum dry matter values have been recognized as international standards for each variety. These values ranged from 19 to 25%, depending on the variety. It is a matter of fact that avocado fruits that are harvested with dry matter levels below the recommended values will ripen irregularly and will fail to reach good quality attributes. Also, avocado fruits that are harvested with a high dry matter will go through a fast ripening and a decreased shelf life. In other words, fruits harvested either premature or over-mature are more vulnerable to postharvest physiological disorders than fruits harvested at the proper maturity state (Kader, 1999). Consequently, to determine the stage of development at which fruit meets minimum acceptable quality, studies that will identify quicker, easier, and more reliable techniques measuring the maturity of individual avocado fruit are therefore crucial for the advancement of avocado industry. The storage cooling system in which fruits are transported also plays an important role and affects the physiochemical conditions (Astudillo-Ordóñez and Rodríguez, 2018). Non-destructive methods such near-infrared have also been used as the indicating parameters, but cannot give a precise maturity stage (Blakey, 2016). More data are needed to improve the accuracy of non-destructive methods, which will undoubtedly save time and money if they can be relied on.

**Figure 1:**
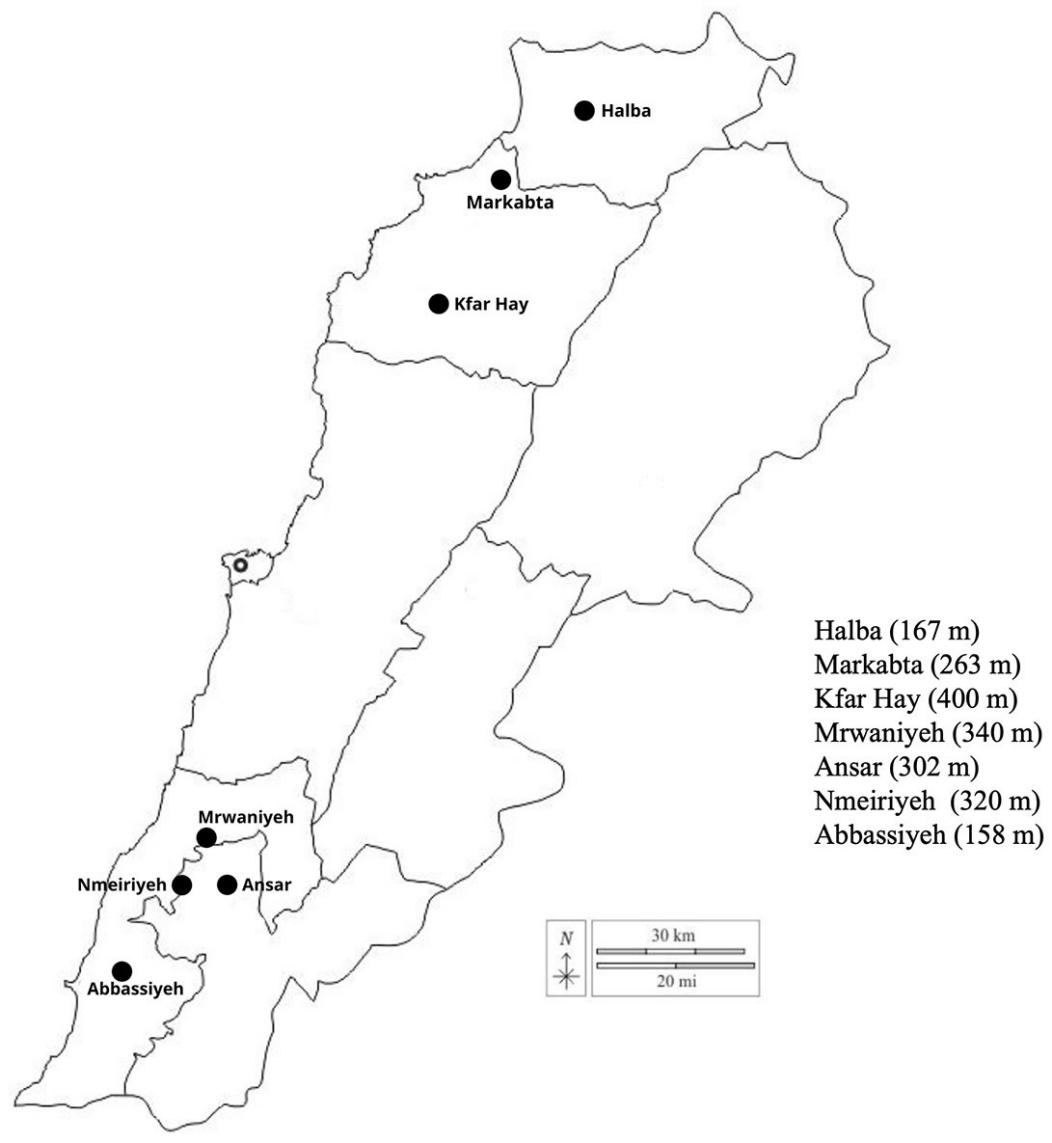
Map of the south (Nmeiriyeh, Marwanieh, Ansar, and Abbasiyeh) and north locations (Halba, Kfar Hay, and Markabta) used in this study and their altitudes.

The primary goal of this study was to assess postharvest maturity indices in commercial avocado varieties in relation to different elevations on Lebanon's coast that are directly related to export market criteria, particularly the European market. Exploring the European market is a perfect alternative to alleviate the agricultural sector's collapse that the country might encounter. The varieties in this study were as follows: Hass, Lambhass, Ettinger, Fuerte, Pinkerton, Reed, and Horshim. The following locations having different altitudes and varying from 50 to 400 m were chosen: Abbasiyeh, Nmeiriyeh, Marwanieh, Ansar, Kfar Hay, Halba, and Markabta as shown in Figure 1.

In this study, avocado maturity indices were calculated from November 2020 to March 2021. Based on the literature and the agricultural organizations' data, this study that encompasses the majority of commercial avocado varieties and their planted sites is being undertaken for the first time in Lebanon. Moreover, avocado cultivation is relatively new in Lebanon, and many orchards were not set up properly leading to the production of non-commercial varieties which results in low productivity rate and profitability impacting micro-, small, and medium enterprises (MSME) livelihoods. As a result, expanding the scope of this study to other areas where avocado is grown under similar conditions could be beneficial to the entire MENA region.

Key industry groups could then use the data to develop maturity-based strategies for improving fruit quality.

## Materials and Methods

Avocado fruits of different varieties were picked by hand from 7 different locations along the coast of Lebanon, either from south (Nmeiriyeh, Marwanieh, Ansar, and Abbasiyeh) or north (Halba, Kfar Hay, and Markabta). Figure 1 shows the map of these locations and their altitudes. These locations vary in temperature, latitude, and the applied agricultural practices. Each orchard was split into column and rows, and each harvested tree was given a unique code. Fruits were harvested from November to March on a 2-week interval. Since the environmental conditions and agricultural practices might influence the change in maturity indices, two Supplementary Data Tables were added to describe this information for each studied location to better link the data with the location's environmental conditions and agricultural practices. Supplementary Table S1 presents the environmental conditions such as temperature, humidity, precipitation, and solar radiation. Supplementary Table S2 represents the main agricultural practices used by the farmers, such as the amount and type of fertilization, pesticides, and irrigation system.

### Plant Material

**Figure 2:**
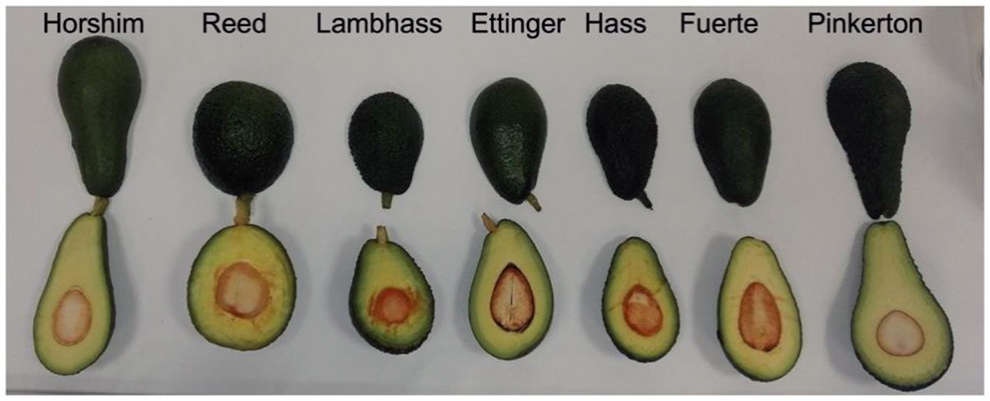
The seven avocado varieties studied in this experiment (Horshim, Reed, Lambhass, Ettinger, Hass, Fuerte, and Pinkerton).

Fruits of the following varieties, Horshim, Fuerte, Pinkerton, Hass, Lambhass, Ettinger, and Reed, were collected. Sampling was performed on at least 10–15% of total tree number in each orchard. In other words, if an orchard has 300 trees, at least 30–40 trees were sampled to represent the varied locations of this orchard. The orchard was split into rows, and fruits were harvested from each row to ensure the representatives of the experiment. A number of 5 to 6 fruits were picked from each tree and coded according to the variety, farm location, farmer name initials, row number, and tree number. Figure 2 represents the avocado varieties used in this study.

The fruits were placed in perforated transparent bags and transferred to the laboratory for postharvest physiochemical analysis. This harvesting process was done regularly every 2 weeks and in the early morning. The avocado samples were visually inspected at the laboratory to ensure that they were not subjected to any damage during transportation and if they were, the damaged ones were excluded from the samples.

### Physiochemical Characteristics

The avocado fruits were subjected to several measurements starting from weighing 4 fruits from each tree individually, measuring their circumference and fruit length using a caliper. Then, the firmness of each fruit was measured 4 times using a penetrometer (the two sides without skin). Firmness was measured after peeling off the skin using an Effegi FT 011 (0–5 kg) penetrometer equipped with an 11-mm plunger. Afterward, each fruit was divided into 2 parts. The first part was labeled and freeze-dried for the oil content quantification, and the other part was used for the dry matter determination. The dry matter analysis was performed using the coring method. Approximately 5 g from each side of the equator of the fruit using a 13.2-mm coring cylinder was removed and then placed in a drying oven at 60°C for 36 h. Fruit samples were dried until a constant weight was achieved, after which the dry matter content was calculated using the standard procedure (Ranney et al., 1992). The results were recorded as percent of the original weight and reported as an average of the four individual values. For oil quantification, fruit samples were ground, and small portions were weighed and then freeze-dried. About 0.5 g of dry sample was weighed in a tared XT4 filter bags (Ankom Technology) and sealed very well. Then, the bags were put in the Soxhlet extractor. A mixture of hexane acetone (1:1) was prepared, and around 300 ml was put in the 500-ml round bottom flask with boiling chips. The setup for Soxhlet extraction was connected (mantle, round bottom flask, extractor, and condenser). The cooler and the heater were turned on. As soon as the solvent started to evaporate, the heat was regulated. After 4 h, the heat was turned off and the bags were removed from the extractor and kept in the hood for few minutes for the excess solvents to dry. Then, the bags were cooled in a desiccator and weighed after 1 h in the oven.

The remaining parts were then blended into juice and stored in a freezer for a later brix (TSS) test by placing one or two drops of the juice on the prism of the digital refractometer (PR-32 α Palette; Atago, Japan). The titratable acidity was measured by diluting 5 ml of the juice with 95 ml of dH_2_O to measure the pH using a standard pH meter. About 0.1 M NaOH was added until a constant pH 8.1 was reached. Titratable acidity was calculated as the number of milliliters of NaOH used multiplied by an appropriate factor using this equation (X^*^0.075^*^0.1)/5) ^*^100, where X is the amount of NaOH used, and 0.075 is the acid milliequivalent conversion factor.

###  Statistical Analysis

After the data were entered and cleaned from extreme values, statistical comparison of all parameters was performed using *t*-test and one-way ANOVA test. Differences were considered statistically significant for *p*-values < 0.05 using RStudio statistical software. Principal component analysis (PCA) was performed over the 3 harvesting stages to observe the clustering behavior of the varieties and correlations between maturity indices. Shapiro–Wilk normality tests and studentized residual plots were used to test error assumptions of variance analysis, including random, homogenous, and normal distribution of error. Means were calculated using the LSMEANS statement, and significant differences between the treatments were determined by the Tukey–Kramer test with α = 0.05 and are mentioned in each figure or table. Statistical comparison of maturity indices under different locations by variety and harvest stage was performed using one-way ANOVA parametric test followed by Student–Newman–Keuls (SNK) *post hoc* test. As a non-parametric alternative to ANOVA, the Kruskal–Wallis test was used followed by Dunn's test. Differences between locations for the same variety were considered statistically significant for *p*-values < 0.05 using RStudio statistical software.

## Results

The weight of avocado fruits was measured for all varieties from all the locations. Quality parameters of all varieties and locations are presented in Table 1. The results showed that the lowest weight was recorded for Hass fruits harvested from Markabta (139.4 g), whereas the highest one was for Ettinger fruits harvested from Marwanieh (450.5 g). All the varieties except Fuerte showed a significant difference in their weight between locations. For Hass avocado, the highest value was recorded in Abbasiyeh (213.1 g), whereas the lowest value was recorded in Markabta (139.4 g). Regarding Lambhass, the highest value was recorded in Marwanieh (277.7 g), whereas the lowest weight was in Abbasiyeh (190.02 g). Pinkerton fruits showed to have a big weight in Marwanieh fruits (346.1 g), whereas the lowest was in Markabta (231.1 g). For Reed avocado, the highest value was recorded in Ansar (320.5 g), whereas the lowest was in Kfar Hay (253.5 g). Concerning Ettinger, the heaviest fruit was obtained in Marwanieh (450.5 g), whereas the lowest weight was for avocado harvested from Ansar (274.3 g).

**Table 1:** Physiochemical characteristics: titratable acidity %, firmness without skin (KgF), total soluble solids %, and weight (g) of the different avocado varieties over the 3 harvesting stages.

**Variety**	**Location**	**TA %**					**FWS (KgF)**					**TSS%**					**Weight (g)**					**Caliber (cm)**					**Lenght (cm)**				
Hass	Abbasiyeh	0.17	±	0.06			18.58	±	0.86			10.2	±	0.8	a		213.1	±	30.6	a		21.17	±	1.03	a		11.32	±	0.73	a	
	Ansar	0.11	±	0.02			17.79	±	1.67			8.46	±	1.05	bc		173.04	±	32.49	b		19.28	±	1.33	b		9.85	±	0.86	c	
	Kfar Hay	0.11	±	0.02		NS	17.03	±	1.76		NS	7.67	±	0.87	d	***	170.38	±	25.46	b	***	19.03	±	0.72	b		9.95	±	0.72	c	***
	Markabta	0.09	±	0.01			18.67	±	1.57			8	±	1.19	cd		139.41	±	19.02	c		17.93	±	0.8	c		9.25	±	0.51	d	
	Mrwaniyeh	0.16	±	0.05			18.17	±	1.3			9.08	±	1.07	b		203.7	±	24.95	a		20.74	±	0.91	a		10.55	±	0.98	b	
	Nmeiriyeh	0.11	±	0.02			18.94	±	1.82			8.53	±	0.78	bc		165.14	±	20.3	b		19.38	±	0.65	b		10.25	±	0.7	bc	
Fuerte	Abbasiyeh	0.11	±	0.04	a		14.5	±	1.06	bc		8.63	±	1.4	ab		291.78	±	46			22.7	±	1.72	ab		12.48	±	0.97	b	
	Ansar	0.12	±	0.01	a		14.8	±	1.32	bc		7.76	±	0.84	bc		298.46	±	45.6			22.13	±	1.29	ab		13.52	±	1.21	ab	
	Kfar Hay	0.1	±	0.03	a		15.55	±	1.33	b		7	±	0.9	c		285.41	±	64.3			21.61	±	1.22	b		13.24	±	1.28	ab	
	Markabta	0.12	±	0.01	a	**	14.6	±	1.65	bc	***	6.7	±	0.56	c	***	334.23	±	57.2		NS	23.21	±	1.18	ab	**	14.48	±	0.99	a	**
	Mrwaniyeh	0.09	±	0.01	b		14.71	±	0.88	bc		8.53	±	1.05	ab		319.94	±	59.18			23.23	±	1.54	ab		12.54	±	1.24	b	
	Nmeiriyeh	0.1	±	0.02	a		16.67	±	1.33	a		7.38	±	0.92	bc		293.09	±	56.34			22.15	±	1.34	ab		13.46	±	1.19	ab	
	Halba	0.12	±	0.02	a		13.83	±	1.16	c		9.33	±	0.41	a		284.25	±	47.8			23.48	±	0.68	a		14.01	±	1.39	a	
Pinkerton	Abbasiyeh	0.13	±	0.04	a		17.44	±	1.76			9.67	±	0.73	a		250.6	±	45	bc		20.57	±	1.31	bc		13.74	±	1.2	b	
	Ansar	0.08	±	0.01	b		18.04	±	1.32			9	±	1.18	ab		274.4	±	45.9	b		21.37	±	1.05	b		14.17	±	1.24	ab	
	Kfar Hay	0.11	±	0.02	ab		17.04	±	1.93			8.44	±	1.12	b		255.14	±	36.4	bc		20.86	±	1.1	bc		14.95	±	1.01	a	
	Markabta	0.1	±	0.02	ab	*	17.67	±	1.62		NS	8.41	±	1.3	b	**	231.1	±	46	c	***	20.07	±	1.31	c	***	14.04	±	1.25	ab	**
	Mrwaniyeh	0.1	±	0.02	ab		18.85	±	0.81			8.9	±	0.87	ab		346.1	±	59.4	a		23.11	±	2.03	a		15.01	±	1.2	a	
	Nmeiriyeh	0.08	±	0.02	b		18.49	±	1.58			8.52	±	0.75	b		317.8	±	54.3	a		22.85	±	1.13	a		13.68	±	0.96	b	
Lambhass	Abbasiyeh	0.18	±	0.03			15.43	±	1.67			10.2	±	0.88	a		190.02	±	25.4	c		20.26	±	1.09	b		9.95	±	0.75	ab	
	Ansar	0.11	±	0.02		NS	15.58	±	1.11		NS	9.02	±	0.94	b	***	225.45	±	28	b	***	22.18	±	0.64	a	***	8.76	±	1.31	b	***
	Mrwaniyeh	0.13	±	0.03			14.92	±	1.5			9.14	±	0.52	b		277.7	±	36	a		23.25	±	1.17	a		10.85	±	1.15	a	
	Halba	0.09	±	0			15.2	±	0.26			9.7	±	0.3	ab		193.46	±	27.14	c		20.5	±	1.5	b		9.56	±	1.06	b	
Ettinger	Ansar	0.08	±	0.01			13.62	±	1.65	ab		8.28	±	1.24			274.3	±	35.8	c		22.24	±	0.87	b		12.15	±	1.26	b	
	Kfar Hay	0.14	±	0.08		NS	9.86	±	1.27	c	***	7	±	0.77		NS	297.6	±	56.4	bc	***	22.88	±	1.28	b	***	13.75	±	1.78	a	***
	Mrwaniyeh	0.11	±	0.03			11.99	±	1.73	b		7.94	±	0.69			450.5	±	71.5	a		25.17	±	1.43	a		14.88	±	1.08	a	
	Halba	0.2	±	0.11			14.16	±	1.8	a		8.02	±	1.09			358	±	101.1	b		23.98	±	2.21	ab		14.08	±	1.57	a	
Reed	Abbasiyeh	0.17	±	0.03	a		13.72	±	1.8	bc		8.85	±	0.78	a		285.7	±	44.5	b		25	±	1.37	b		9.51	±	0.67		
	Ansar	0.1	±	0.03	b	***	15.06	±	1.4	a	***	8.07	±	0.56	b	***	320.5	±	61	a	***	26.7	±	1.83	a	***	9.8	±	1.41		NS
	Kfar Hay	0.12	±	0.06	b		13.2	±	1.25	c		7.6	±	1.02	b		253.5	±	41	c		22.75	±	1.11	c		9.66	±	0.68		
	Nmeiriyeh	0.1	±	0.02	b		14.16	±	1.21	b		8.09	±	0.63	b		266.4	±	43.2	bc		24.56	±	0.83	b		9.73	±	0.53		
Horshim	Ansar	0.16	±	0.05			15.2	±	2.1			7.98	±	1.02			315.5	±	33.15			22.3	±	1.03			14.38	±	1.07		

Mean values ± standard deviation followed by the same letter are not significantly different at p ≤ 0.05 level. *P < 0.05; **P < 0.01; ***P < 0.001.

The caliber of avocado fruits was measured for all varieties from all the locations. The results showed that the lowest circumference was recorded for Hass fruits harvested from Markabta (17.93 cm), whereas the highest one was for Reed fruits harvested from Ansar (26.7 cm). All the varieties showed a significant difference in their circumference between locations. For Hass avocado, the highest value was recorded in Abbasiyeh (21.17 cm), whereas the lowest value was recorded in Markabta (17.93 cm). Regarding Lambhass, the highest value was recorded in Marwanieh (23.25 cm), whereas the lowest circumference was in Abbasiyeh (20.26 cm). Pinkerton fruits showed to have a big caliber in Marwanieh fruits (23.11 cm), whereas the lowest was in Markabta (23.07 cm). For Reed avocado, the highest value was recorded in Ansar (26.70 cm), whereas the lowest was in Kfar Hay (22.75 cm). Concerning Ettinger, the fruit with the highest caliber was in Marwanieh (25.17 cm), whereas the lowest one was for avocado harvested from Ansar (22.24 cm).

The length of avocado fruits was measured for all varieties from all the locations. The results showed that the lowest length was recorded for Lambhass fruits harvested from Ansar (8.76 cm), whereas the highest one was for Pinkerton fruits harvested from Marwanieh (15.01 cm). All the varieties showed a significant difference in their length between locations except Reed. For Hass avocado, the highest value was recorded in Abbasiyeh (11.32 cm), whereas the lowest value was recorded in Markabta (9.25 cm). Regarding Lambhass, the highest value was recorded in Marwanieh (10.85 cm), whereas the lowest length was in Ansar (8.76 cm). Pinkerton fruits showed to have a large length in Marwanieh fruits (15.01 cm), whereas the lowest was in Nmeiriyeh (13.68 cm). For Reed avocado, the highest value was recorded in Ansar (9.80 cm), whereas the lowest was in Abbasiyeh (9.51 cm). Concerning Ettinger, the fruit with the highest length was in Marwanieh (14.88 cm), whereas the lowest one was for avocado harvested from Ansar (12.15 cm).

The firmness without skin of the avocado varieties was studied and compared between different locations. The highest firmness value was recorded for Hass and Pinkerton avocado harvested from Nmeiriyeh and Marwanieh, respectively (18.9 Kgf), whereas the lowest one was shown for Ettinger fruits from Kfar Hay (9.86 Kgf).

The values for Fuerte, Ettinger, and Reed showed a significant difference among the studied locations. Regarding Fuerte, fruits in Nmeiriyeh showed the highest firmness value (16.7 KgF) that was significantly different from all other locations including Halba which showed the lowest value (13.8 KgF). Ettinger avocado fruits had their minimum firmness in fruits harvested from Kfar hay (14.2 KgF) that was significantly different from all other locations including Halba which showed the highest value (9.86 KgF). For Reed variety, the highest firmness was recorded in Ansar (15.1 KgF) which was significantly different from all other locations including Kfar Hay which showed the lowest value (13.2 KgF).

### Dry Matter (DM)

**Figure 3:**
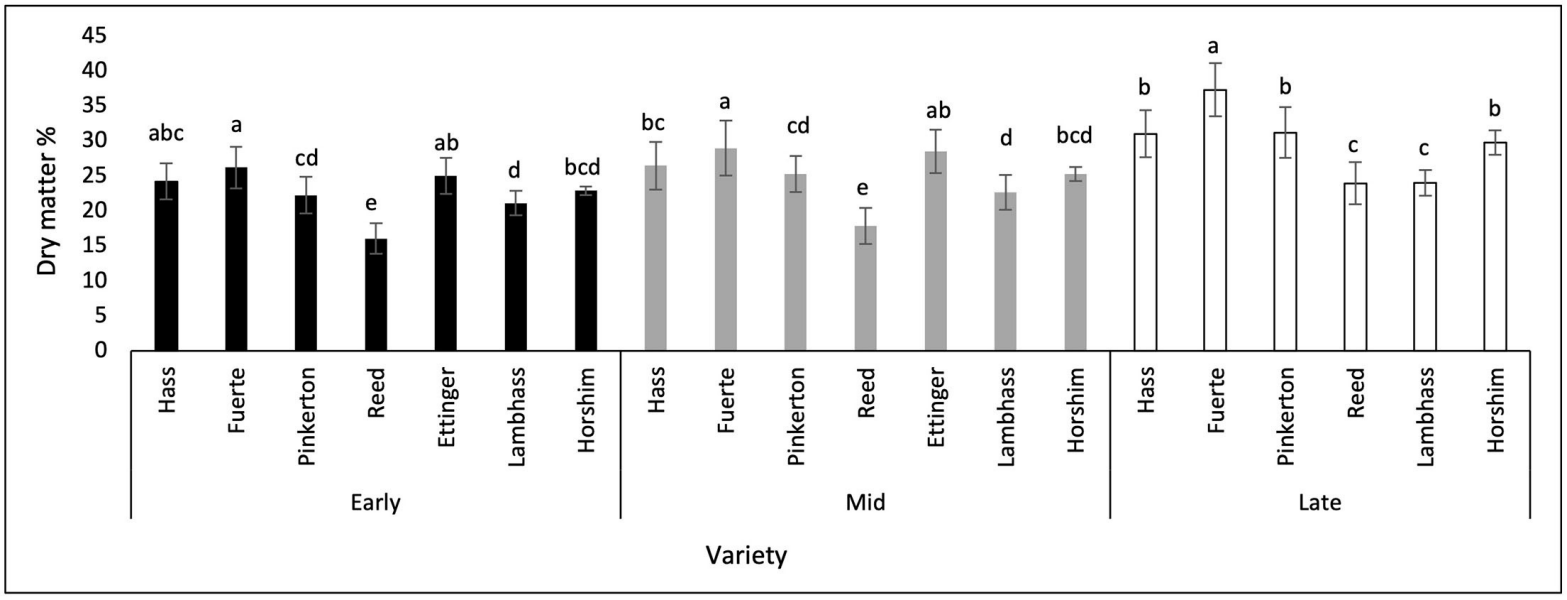
A graph showing the dry matter % of different avocado varieties through three harvesting stages. Mean values followed by the same letter are not significantly different at *p* ≤ 0.05 level. Variables were considered significant < 0.05 risk level.

The dry matter percentage of each variety was measured throughout the season from November 2020 till March 2021. As shown in Figure 3, during the early harvest period which is from November till early December 2020, the highest dry matter percentage was in Fuerte (26.22%) shared with Ettinger and Hass, whereas the lowest one was recorded in Reed (16.08%). During this stage, a significant difference in DM% was observed between Reed and all other varieties.

During the mid-harvest stage also, which extends from late December till January 2021, Fuerte shared with Ettinger, Hass, and Pinkerton showed the highest DM values (28.99%), whereas Reed showed the lowest (17.86%). During this period, Reed fruits dry matter had a significant difference from all other varieties' DM percentages.

During the late harvest stage, Fuerte fruits also had the highest dry matter percentage among other varieties (37.30%), where Ettinger fruits were not included during this stage due to their earlier harvest period, thus they were not available for sampling. The lowest DM% was obtained in Reed (23.97%) shared with Lambhass (24.06%). At this period of harvest, it was obvious that Fuerte dry matter % showed a significant difference from all other varieties. Horshim, Hass, and Pinkerton showed no significant difference with each other nor did Lambhass and Reed. However, the two groups of varieties showed a significant difference with each other and with Fuerte fruits.

When comparing the 3 harvesting stages together, it was shown that a significant difference in DM was seen in Reed, Pinkerton, and Lambhass between the late harvest stage and the previous stages. This reflects a significant increase in the dry matter percentages when the fruits reach the late stage where Reed increased by 7%.

**Figure 4:**
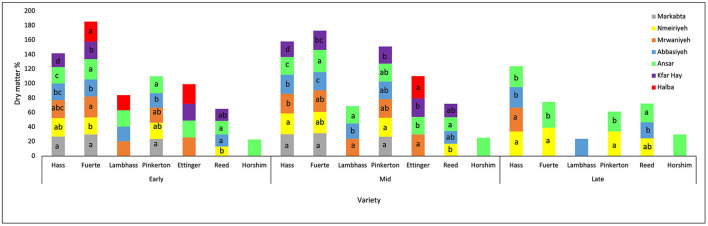
A graph showing the dry matter % of different avocado varieties from different locations through three harvesting stages. Mean values followed by the same letter are not significantly different at *p* ≤ 0.05 level. Variables were considered significant < 0.05 risk level.

When comparing the dry matter percentage of the varieties throughout the three harvesting stages, it was shown that Reed avocado increased by 33% from the early to the late stage. However, Lambhass dry matter increased by only 12%. The remaining varieties had a moderate increase ranging from 22 to 28%. When studying the dry matter during the season depending on the location of the fields by two-way ANOVA, it was shown in Figure 4 that during the early harvest stage, Fuerte that showed the highest DM values had no significant difference in DM of Markabta, Marwanieh, Ansar, and Halba, nor did Kfar hay, Abbasiyeh, and Nmeiriyeh. However, the two groups of locations together showed a significant difference. Regarding Pinkerton, the dry matter % in Abbasiyeh showed a significant difference with Markabta and Ansar. This was the case also with Hass fruits, in addition to a significant difference in Kfar hay's DM % with all other locations. Measurements of Reed fruits showed that the DM % in Nmeiriyeh had a significant difference from that of Abbasiyeh and Ansar. The remaining varieties showed no significant difference in the DM % between the locations.

During the mid-harvest stage, a significant difference between locations of all varieties was observed. Regarding Reed variety, a significant difference was detected between the dry matter percentage of Nmeiriyeh and Ansar. Kfar hay and Ansar did not show a significant difference, nor Halba and Marwanieh for Ettinger avocado. However, the DM% of the two groups of locations were significantly different. For Pinkerton variety, the DM in Markabta and Nmeiriyeh showed a significant difference from that of Kfar hay. Concerning Lambhass, the DM% of Abbasiyeh is significantly different from both Ansar and Marwanieh. For Fuerte, the dry matter of Marwanieh, Nmeiriyeh, Markabta, and Ansar has a significant difference from that Kfar hay and Abbasiyeh. Nmeiriyeh and Markabta showed no significant difference for Hass fruits, nor did Marwanieh and Abbasiyeh. However, the 2 groups show significant differences with each other and the remaining varieties. During the late harvest stage, the locations were limited where not all the fields were used. A significant difference was observed between the dry matter of Nmeiriyeh and Ansar in both Fuerte and Pinkerton. Regarding Hass fruits, the DM% in Nmeiriyeh and Marwanieh showed no significant difference nor did Ansar and Abbasiyeh. However, the two groups together had a significant difference. For Reed avocado fruits, the dry matter in Ansar and Abbasiyeh showed a significant difference.

### Oil Content

**Figure 5:**
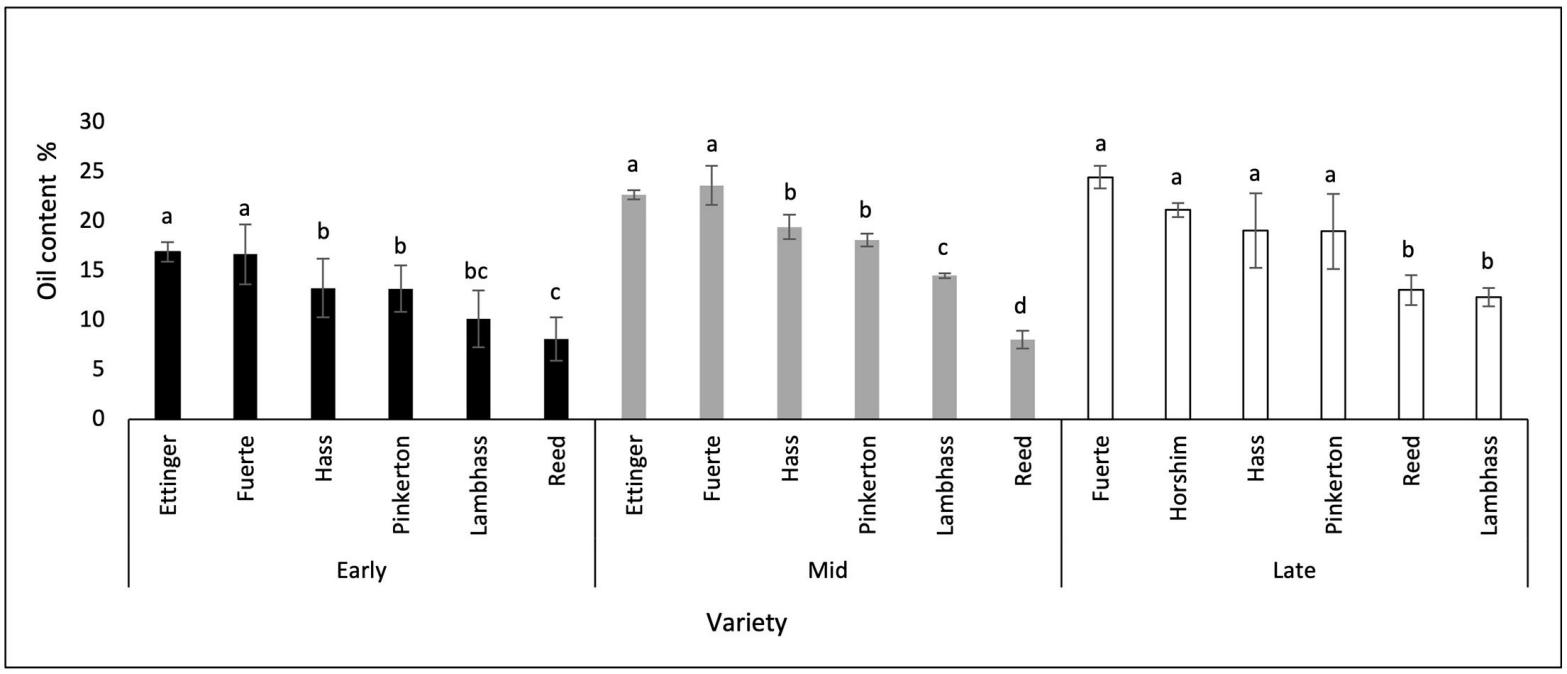
A graph showing the oil content % of different avocado varieties through three harvesting stages. Mean values followed by the same letter are not significantly different at *p* ≤ 0.05 level. Variables were considered significant < 0.05 risk level.

The oil content percentage of avocado varieties was also measured throughout the harvesting stages from late November 2020 till March 2021 (Figure 5). During the early harvest stage which extended from late November till December, Ettinger avocado shared with Fuerte had the highest OC % (16.945 and 16.69143%, respectively). The lowest OC% was shown in Reed fruits which was 8.118%. The significant difference between the OC% of the studied varieties was obtained where Ettinger and Fuerte showed no significant difference in OC % nor did Hass, LambHass, and Pinkerton. However, the OC of the two groups together was significantly different. Moreover, Reed avocado fruits showed a significant difference from all studied varieties other than Lambhass.

During the mid-harvest stage, Fuerte fruits showed the highest oil content % (23.6525%) shared with Ettinger (22.715%), whereas the lowest OC value was observed for Reed fruits (8.05%). When studying the significant difference in OC % between the varieties, it was shown that Ettinger and Fuerte were not significantly different nor were Hass and Pinkerton. However, the two groups' OC% together had a significant difference with each other and with Reed and Lambhass as well.

For the late harvest stage, Fuerte showed the highest oil content value (24.48%), where Ettinger fruits were not included during this stage due to their earlier harvest period, thus they were not available for sampling, whereas the lowest OC% was observed for LambHass avocado (12.355%) shared with Reed (13.08%). Regarding the differences in OC% between the studied varieties, it was shown that Fuerte, Hass, Horshim, and Pinkerton had no significant difference in their oil content percentage nor did Lambhass and Reed. However, the two groups' OC% was significantly different when comparedto eachother.

When observing the oil content % results throughout the 3 harvesting stages altogether, it was observed that the oil content % of Fuerte, Hass, Pinkerton, and Reed avocado fruits had increased by around 31% from the early to the late harvest stage. However, Lambhass fruits showed around 18% increase in OC % throughout the 3 harvesting stages.

**Figure 6:**
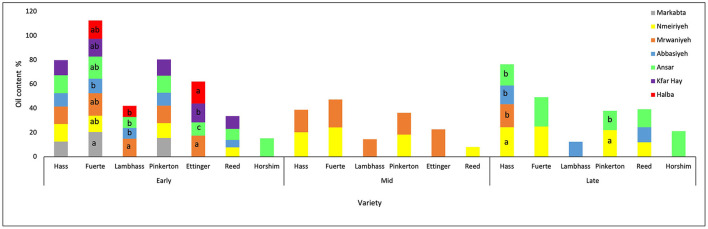
A graph showing the oil content % of different avocado varieties from different locations through three harvesting stages. Mean values followed by the same letter are not significantly different at *p* ≤ 0.05 level. Variables were considered significant < 0.05 risk level.

During the early harvest stage, the difference in oil content of the avocado varieties between locations was studied (Figure 6) and it was observed that Halba and Ansar had a significant difference in OC between each other and with all the other locations for Ettinger fruits. Regarding Fuerte, Markabta, and Abbasiyeh, they had a significant difference in their OC%. The oil content in Lambhass is significantly different between Marwanieh and the other locations. There was no significant difference concerning the remaining varieties.

During the mid-harvest stage, only 2 harvesting locations were studied. However, no significant difference was observed in the oil content of all varieties. During the late harvest stage, a significant difference in OC% was observed for Hass fruits where Nmeiriyeh showed a significant difference with all other locations. The OC% of Pinkerton fruits also showed a significant difference between Ansar and Nmeiriyeh. The other varieties' OC differences were not significant.

The total soluble solid (TSS) of avocado fruits was measured for all varieties from all the locations. The results showed that the highest TSS was recorded for Hass and Lambhass fruits harvested from Abbasiyeh (10.2%), whereas the lowest one was for Fuerte fruits harvested from Markabta (6.7%). All the varieties except Ettinger showed a significant difference in their TSS between locations. For Hass avocado, the highest value was recorded in Abbasiyeh (10.2%), which was significantly different from all other locations including Kfar hay which showed the lowest brix value (7.67%). Regarding Lambhass, the highest value was recorded in Abbasiyeh (10.2%), whereas the lowest one was in Ansar (9.02%). Pinkerton fruits showed to have the highest brix % in Abbasiyeh fruits (9.67%), whereas the lowest was in Markabta (8.41%). For Reed avocado, the highest value was recorded in Abbasiyeh (8.85%), which was significantly different from all other locations including Ansar which showed the lowest TSS value (8.07%). Concerning Fuerte, the fruit with the highest brix % was in Halba (9.33%), whereas the lowest one was for avocado harvested from Markabta (6.7%).

The titratable acidity of the avocado varieties was studied and compared between different locations. The highest acidity value was recorded for Lambhass avocado harvested from Abbasiyeh (0.18%), whereas the lowest one was shown for Ettinger fruits from Ansar, and Pinkerton harvested from Ansar and Nmeiriyeh (0.08%). The values for Fuerte, Pinkerton, and Reed showed a significant difference among the studied locations. Regarding Fuerte, fruits in Marwanieh showed the lowest TA value (0.09%) that was significantly different from all other locations including Ansar, Markabta, and Halba which showed the highest values (0.12%). Pinkerton avocado fruits had their maximum TA% in fruits harvested from Abbasiyeh (0.13%), whereas Nmeiriyeh and Ansar showed the lowest values (0.08%). For Reed variety, the highest TA% was recorded for Abbasiyeh (0.17%), whereas the lowest was in Nmeiriyeh and Ansar (0.1%).

### Principal Component Analysis (PCA)

A PCA was done for the avocado fruits to study the correlation between the maturity indices throughout the harvesting season. A number of three principal component analyses were performed for the avocado fruits to study the correlation between the maturity indices and to observe the clustering behavior of the varieties throughout the 3 harvesting stages in the season.

**Figure 7:**
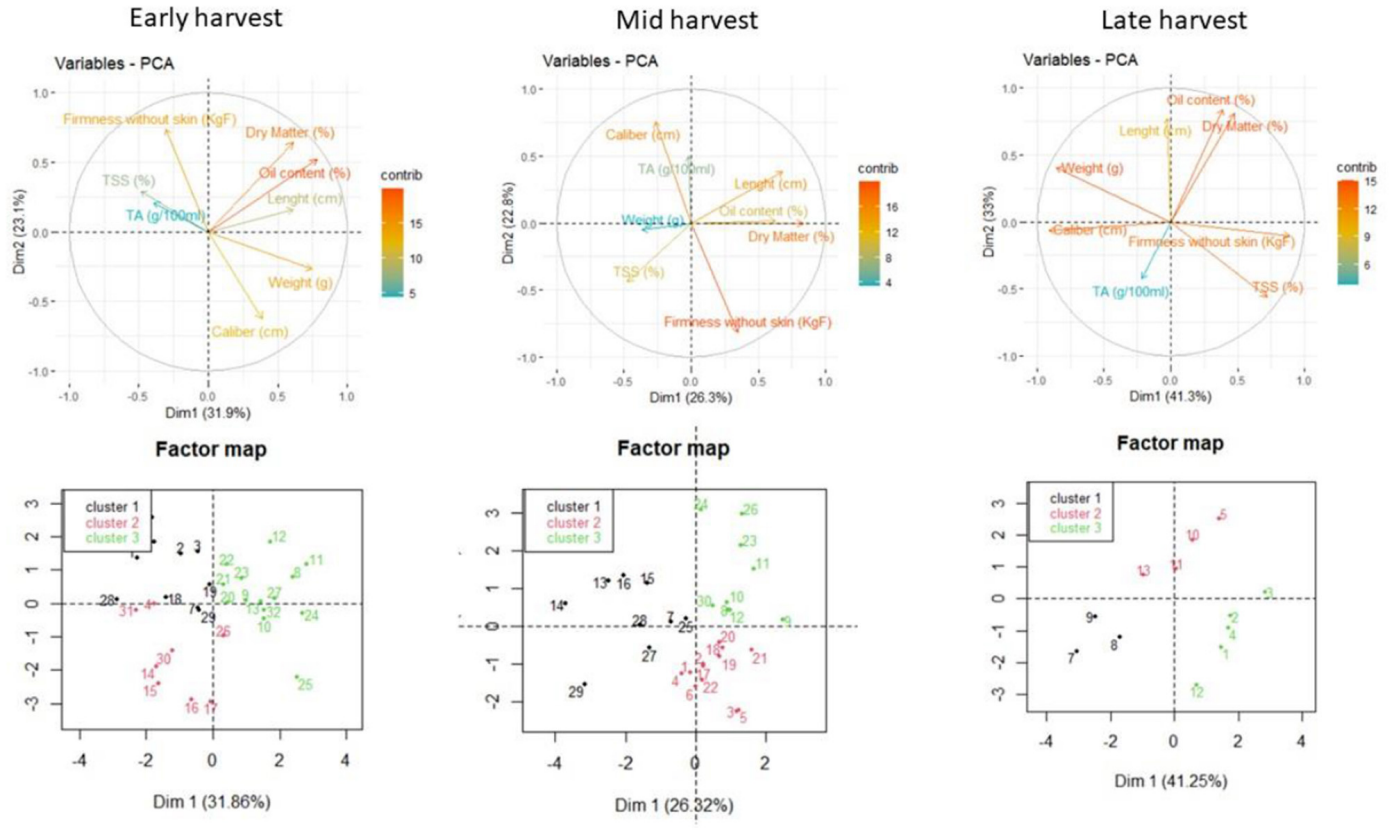
PCA graphs showing the correlation and clustering behavior of the studied maturity indices.

As shown in Figure 7, during the first harvesting stage, a strong correlation was observed between the dry matter % and the oil content (r^2^ = 0.77) and a moderate positive correlation between the caliber (cm) and weight (g) (r^2^ = 0.59).

The 1st principal component was positively correlated with the variables oil content (%), weight (g), dry matter (%), and length (cm) and negatively correlated with the TSS (%) and TA (%). The 2nd principal component had a positive correlation with dry matter % and firmness without skin (KgF) and a negative correlation with weight (g) and caliber (cm).

The factor map of the early harvesting stage allowed the detection of classes or groups of individuals associated with maturity indices as per Figure 7. Cluster 1: high TSS (%), firmness without skin (KgF) and TA (%), low weight (g), and caliber (cm); Cluster 2: low firmness without skin (KgF), dry matter (%), and oil content (%); Cluster 3: high dry matter (%), oil content (%), and weight (g).

During the mid-harvest stage, the correlation between the oil content (%) and dry matter (%) tended to get stronger (r^2^ = 0.98). There is a positive correlation between caliber (cm) and weight (g) (r^2^ = 0.69).

The 1st principle component was positively correlated with the variables dry matter (%), length (cm), and oil content (%) and moderately negatively correlated with the TSS (%). The 2nd principle component had a positive correlation with the caliber (cm) and TA (%) and negative correlation with firmness without skin (KgF) and TSS (%).

The factor map of the mid-harvesting stage allowed the detection of classes or groups of individuals associated with maturity indices as per Figure 7.

- Cluster 1: high caliber (cm), low firmness without skin (KgF).
- Cluster 2: high firmness without skin (KgF), low caliber (cm).
- Cluster 3: high dry matter (%), oil content (%), and length (cm), low TSS (%).

During the late harvest stage, the correlation between the dry matter % and oil content % of the avocado fruits was still strong (r^2^ = 0.95). The weight showed a negative correlation with firmness without skin (r^2^ = −0.72) and the TSS% (r^2^ = −0.74). The TSS% was positively correlated with the firmness without skin (r^2^ = 0.71), and the caliber (cm) had a moderate correlation with weight (g) (r^2^ = 0.59).

The 1st principal component was positively correlated with firmness without skin (KgF) and TSS (%) and negatively correlated with the weight (g) and caliber (cm). The 2nd principal component had a positive correlation with dry matter (%), oil content (%), and length (cm) and a negative correlation with TSS (%).

The factor maps of the three harvesting stages allowed the detection of classes or groups of individuals associated with maturity indices as per Figure 7.

- Cluster 1: high TA (%), low dry matter (%), and oil content (%).
- Cluster 2: high dry matter (%),oil content (%), and low TA (%).
- Cluster 3: high firmness without skin (KgF), TSS (%), and low weight (g).

### Radar Data Interpretation

**Figure 8:**
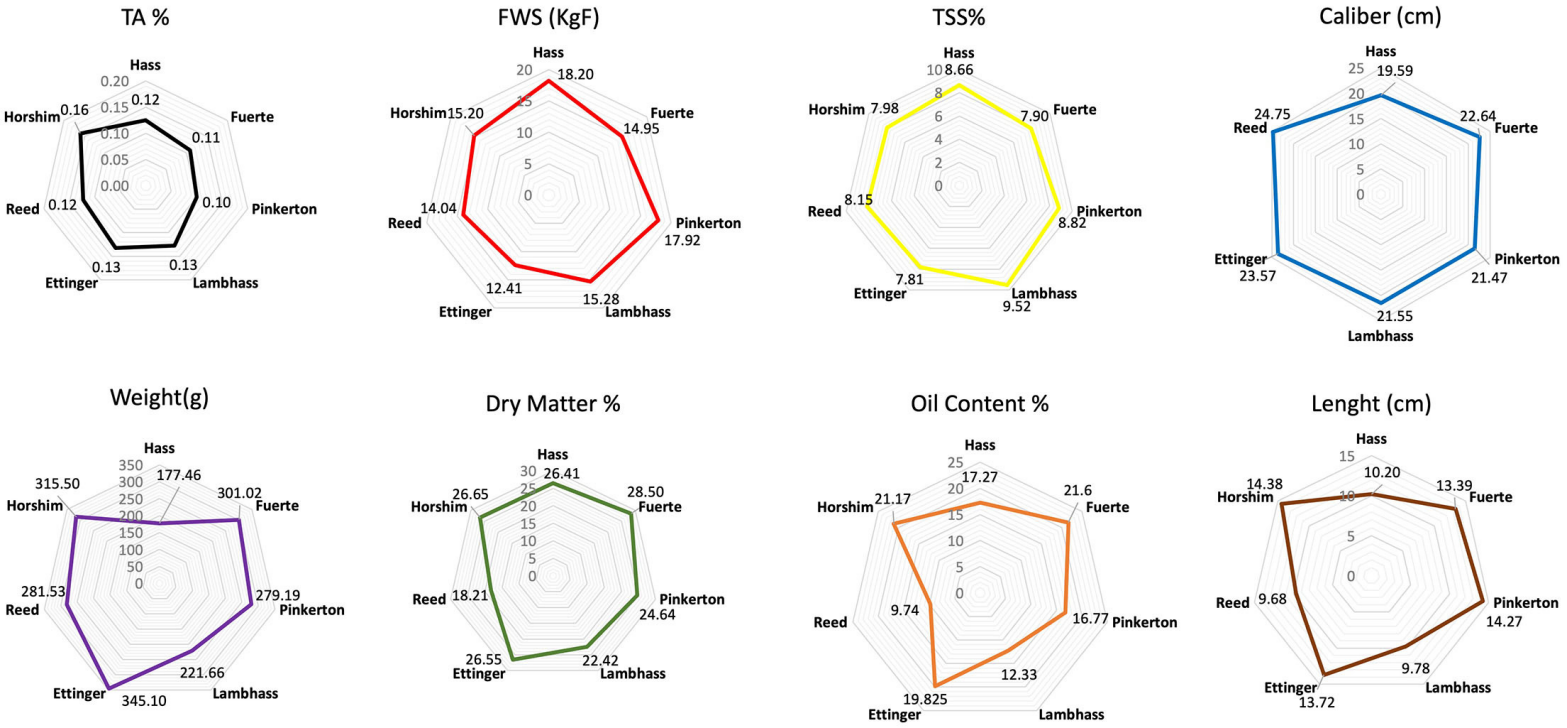
Radar showing the behavior of each maturity indices according to the different avocado varieties.

A radar chart (Figure 8) for every maturity index was done to compare and visualize the behavior of all avocado varieties throughout the whole season. Regarding the titratable acidity, the percentage varied between 0.10% for Pinkerton and 0.16% for Horshim. The other varieties ranged between 0.11 and 0.12% (Fuerte, Reed, Hass, Ettinger, and Lambhass in increasing order).

The firmness without skin of the avocado fruits showed a variation among the varieties where Ettinger had the lowest value (12.41 KgF), whereas the firmest variety was Hass (18.20 KgF). The other varieties' firmness ranged between 14.04 and 17.92 KgF (Reed, Fuerte, Horshim, Lambhass, and Pinkerton in increasing order).

Concerning the total soluble solid percentage (TSS%), the values ranged from 7.81% for Ettinger to 9.52% for Lambhass. The remaining varieties varied between 7.90 and 8.82% (Fuerte, Horshim, Reed, Hass, and Pinkerton in increasing order).

Fruit weight (g) showed an obvious variation among varieties with Ettinger being the heaviest (345.1 g) and Hass being the lightest (177.46 g). The rest of the varieties showed approximately uniform values between 211.66 and 315.5 g (Lambhass, Pinkerton, Reed, Fuerte, and Horshim ascendingly).

Regarding the dry matter, Reed variety had the lowest percentage with 18.21%, whereas Fuerte had the highest dry matter content (28.5%). The other varieties had their dry matter ranging between 22.42 and 26.65% (Lambhass, Pinkerton, Hass, Ettinger, and Horshim in ascending order).

A clear difference in oil content was observed between the studied varieties with Reed having the lowest value (9.74%) and Fuerte having the highest value (21.6%). The other OC% values ranged from 12.33 to 19.825% (Lambhass, Pinkerton, Hass, Ettinger, and Horshim in increasing order).

Hass variety had the lowest fruit caliber (19.59 cm) whereas Reed variety had the highest fruit caliber (24.75 cm). The rest of the varieties showed approximately uniform values between 21.47, 21.55, 22.3, 22.64, and 23.57 cm (Pinkerton, Lambhass, Horshim, Fuerte, and Ettinger ascendingly).

Reed variety had the smallest fruit length (9.68 cm) whereas Horshim variety had the highest length value (14.38 cm). The rest of the varieties showed values between 9.78, 10.2, 13.39, 13.72, and 14.27 cm (Lambhass, Hass, Fuerte, Ettinger, Pinkerton, and Horshim ascendingly).

## Discussion

The avocado production system in Lebanon currently lacks a validated technological model, which increases production uncertainty. Fruit production must ensure the delivery of a high-quality product to the market that meets importing countries' international standards, taking into account agronomic factors such as cultivation, harvest, postharvest, logistics, storage, and marketing. As a result, one of the primary goals of this research is to identify specific parameters that ensure the production of high-quality fruit to position Lebanon and its avocado production in global markets.

In this study, the physicochemical parameters were measured to characterize the initial quality and to determine the best harvest time of seven avocado varieties growing in multiple locations that vary in their altitudes. Previous studies reported that the physiochemical characteristics of “Hass” and “Fuerte” avocado may vary because of the variations in climatic conditions, e.g., change in rainfall and temperature levels (Kaiser et al., 1992; Landahl et al., 2009; Donetti and Terry, 2014).

Our data did not show a significant correlation between fruit weight and orchard's altitude. For instance, the orchard with the smallest Hass fruits (139.4 g) was Markabta located at the one of the highest altitudes (263 m) from the north location, while the biggest Hass weight value (213.1 g) was observed at Abbasiyeh, one of the south locations at 158-m altitude. In contrast, Fuerte fruit weight did not show any significant differences between locations with the highest value recorded in Markabta (334.2 g). This observation is in agreement with Waissbluth and Valenzuela (2007) who reported that fruit maturity in Chile is more related to altitude than to north/south orientation. According to Ferreyra and Defilippi (2012), orchards on Chile's coast take 55 days longer to reach 23%dry matter than those in the central valley zone, owing to climatic conditions and, most likely, agronomic practices (nutrition, pruning, orchard age, and orchard density). Nevertheless, Carvalho et al. (2014) observed a larger fruit size and diameter for higher and medium altitudes in “Hass” avocados in Mexico. Given the fact that in our study, no direct relationship was found between fruit weight and altitudes, more evaluations and factors must be considered such as agricultural practices, crop management, environmental conditions, and others. Considering that the firmness is a very reliable parameter to check the ripening of the fruit (White et al., 1999), it was measured without skin among the studied locations where the firmest varieties were Hass and Pinkerton from south Lebanon (18.9 KgF), this seemed to be higher than what was mainly found in the literature for avocado which could be a reason of the climate and agricultural practices, and the date of harvest as well (Arzate-Vázquez et al., 2011). Based on Kokawa et al. (2020), avocado fruit firmness can be classified into a 4-category scale ranges: soft (0–65 N), medium-soft (66–130 N), medium-hard (131–195 N), and hard (196–300 N). Thus, most of the fruits at the different harvested dates was falling in the medium-hard category. However, the softest variety was Ettinger harvested from the north (9.86 Kgf). The variation among locations for the firmness was obvious for several varieties which followed the same trend of softer fruits from the north. As expected, the hardness of avocado fruits tends to decrease throughout the season (Vallejo-Pérez et al., 2015).

The increase in the dry matter is closely associated with avocado fruit maturation and the ripening process (Kassim and Workneh, 2020). As the season progressed, the dry matter of all varieties in all the locations was increased but in a different rate. Throughout the entire season, Lambhass experienced the lowest increase rate of only 3%, whereas Fuerte experienced the highest increase rate of approximately 11%. To the best of our knowledge, this is the only study that includes such a wide range of varieties and locations. As a result, comparing the data to the literature is more challenging. All of the locations included in this study obtained dry matter percentages >18% at early harvest, except the Reed variety, which registered a range between 13.1 and 18.8%. The differences in the dry matter observed between the orchards were due to the difficulty in visually recognizing the physiological maturity stage of fruits in the field, which does not mean that each orchard cannot achieve the same percentage of dry matter in the fruits. According to Donetti and Terry (2014), the dry matter increased with maturity, regardless of the growing area. Furthermore, in this study, fruit with similar dry matter but from different orchard geographical locations ripened at different rates which vary from 7 to 21 days (data not shown). As a result, segregation of DM into faster or slower ripening fruit might be achieved per a single orchard location, but not segregation for absolute ripening time, because of the orchard-to-orchard difference.

Fruit ripening results in an increase in oil content as well as a decrease in moisture content (Osuna-García et al., 2010). The percentage of oil in the fruit is directly proportional to the percentage of dry matter, allowing the latter to be used as a maturity indicator (Carvalho et al., 2014). Starting in 1925, the California Avocado Industry in the United States used a minimum standard of 8% oil content in the pulp of avocado fruits, but in the 1980s, they began using minimum oil content percentages for each cultivar: 10.0% for Fuerte and 11.2% for Hass (Carvalho et al., 2015). Many studies considered that avocado oil content could be served as a good indicator of fruit maturity (Kassim et al., 2013). As the fruit matures, the concentration of oil within the mesocarp increases as described by Kassim et al. (2013). This increase in oil results in a reduction in the water by the same amount within the fruit implying that the percentage of total water plus oil remains constant throughout the avocado life (Kassim et al., 2013). The minimum oil content necessary for marketing avocado fruit is 8%. After maturation, values >20% can occur. These values occur in the period between harvesting, when commercial maturity is reached, and full maturation, when the oil content increases, and change occurs in the oil composition. Our data show that oil content is variety-dependent and ranged from 8% in Reed to 24.2% in Fuerte. No distinct correlation could be drawn in our study between the effect of altitudes on the percentage of dry matter and oil content of the different avocado varieties. This could be due to multiple factors, such as the variety, agro-ecological conditions of growth, and the fruit development stage (Carvalho et al., 2014). On the other hand, a positive correlation between dry matter and oil content is strongly confirmed by the PCA models throughout the entire growing season. This correlation was mentioned in many previous studies (Snijder et al., 2003). However, Hofman et al. (2000) suggested that the percentage of oil content and dry matter are not the suitable indicators of fruit maturity in late harvested Hass due to the inconsistent physiological changes in late season. Obenland et al. (2012) made the same observation when they concluded that neither dry matter nor oil percentage is sufficient to fully explain the differences in avocado-eating quality; additional means of assessing eating quality would be desirable. As a consequence, the concept of the suitable maturity and use of fruits from low-altitude orchards during the early season and from high-altitude orchards during the late season need to be revised for many consecutive years to take into consideration several factors including the agricultural practices, weather conditions, and disease management. In general, few studies have looked at the impact of altitude on fatty acid quantity and quality. As a result, it has been discovered that the concentration of fatty acids rises with altitude (Carvalho et al., 2015). In this regard, under these environmental conditions, it is possible to increase fruit size percentages, which will improve extra quality percentages, pulp yield, and nutrient content, among other things (Bernal, 2016). However, under these environmental conditions, the limitations of lower yields and longer production cycles must be considered.

The total soluble solid (TSS) of avocado fruits was measured for all varieties from the studied locations. The results showed that the highest TSS was recorded for Hass and Lambhass (10.2%), whereas the lowest one was for Fuerte (6.7%). This contradicts to what was found where Fuerte avocado had a higher TSS range than Hass (Olarewaju, 2014). There was a significant difference in the TSS values between different locations and elevations, and this was also the case with Olarewaju in 2014. The values of the brix for all varieties did not show a consistent trend as expected according to Bertling and Bower (2006). However, in our study, the TSS showed fluctuation throughout the season which was also obtained by Olarewaju in 2014 leading to consider the TSS not suitable for maturity quantification. Therefore, the case with avocado cannot follow the rule of sugar accumulation throughout the growing season as the case of other crops including cherries, kiwifruit, and certain grapes cultivars (Olarewaju, 2014). The titratable acidity of the avocado in different cultivars was studied and compared among the studied locations. The highest acidity value was recorded for Lambhass harvested from Abbasiyeh of south Lebanon (0.18%), whereas the lowest one was shown for Ettinger, and Pinkerton fruits harvested from Ansar and Nmeiriyeh (0.08%). Regarding this parameter, a significant difference was obtained between locations of different elevations where the most acidic fruits were harvested from low altitude locations (Abbasieh, 158 m), and the lower acidic fruits were mainly from high altitude locations (Ansar, 302 and Nmeiriyeh, 320 m). This difference in TA and the effect of elevation can also be observed with other crops including olive fruits in which higher acidity was shown in lower altitude locations (Akça Uçkun and Aksoy, 2020), whereas it shows the opposite trend for other fruits such as citrus (Rokaya et al., 2016). The decrease in TA of ripened fruit may be due to the consumption of organic acid during respiration as the fruit ripens and increasing its pH. It has been suggested that during storage, fruits utilize organic acids for metabolic activities, and this results in a decrease in the TA content during the storage periods which is similar to the present findings. The values for other varieties also showed a significant difference between locations in the same variety. Note that the differences in TA% among locations could also be due to the agricultural practices and climate in addition to the elevation which also applies to different crops such as pineapple (Dorey et al., 2016). Note that it is considered challenging to compare the TA results with literate because this parameter is more common for other types of fruits than avocado. The difference between locations might be due to the difference in environmental location, maturity stage, and harvesting season. According to Hernández-Muñoz et al. (2006), the total acidity is a measure of the organic acid content.

To our knowledge, this is the first study that took into account the effect of geographical locations on ripening and physicochemical characteristics for optimum harvest time per location and in the majority of commercial avocado varieties. This research will be repeated over several seasons to confirm the findings and account for seasonal variations in weather and precipitation rate. This will allow for a better understanding of correlations between all maturity indices, particularly dry matter and oil content values, as well as a recommendation for the best time to harvest during the maturity phase.

## Data Availability Statement

The original contributions presented in the study are included in the article/Supplementary Material, further inquiries can be directed to the corresponding author/s.

## Author Contributions

MS, DN, GL, IR, and WE: conceptualization and investigation. MS, DN, GL, and WE: data curation. MS, DN, and WE: formal analysis and writing—review and editing. IR and GL: funding acquisition and resources. DN, WE, IR, and GL: methodology. IR, GL, and WE: project administration. DN and WE: software and validation. WE: supervision. All authors contributed to the article and approved the submitted version.

## Funding

This work was made possible through the framework and contribution of the Strengthening Exports of Fruits and Vegetables from Lebanon to European and Regional Markets project, which was funded by the Kingdom of the Netherlands and implemented by the René Moawad Foundation (RMF). RMF is a non-profit organization whose mission is to promote Lebanon's long-term social, economic, and rural development.

## Conflict of Interest

GL and IR were employed by René Moawad Foundation. The remaining authors declare that the research was conducted in the absence of any commercial or financial relationships that could be construed as a potential conflict of interest.

## Publisher's Note

All claims expressed in this article are solely those of the authors and do not necessarily represent those of their affiliated organizations, or those of the publisher, the editors and the reviewers. Any product that may be evaluated in this article, or claim that may be made by its manufacturer, is not guaranteed or endorsed by the publisher.

## References

[B1] Akça Uçkun A. Aksoy U. (2020). Effect of yield and quality on olive and olive oil in olive orchards located at different altitudes. Act. Sci. Agri. 4, 33–42. 10.31080/ASAG.2020.04.0853

[B2] Arzate-Vázquez I. Chanona-Pérez J. J. Perea-Flores M. de J. Calderón-Domínguez G. Moreno-Armendáriz M. A.. (2011). Image processing applied to classification of avocado variety hass (Persea americana Mill.) during the ripening process. Food Bioproc. Technol. 4, 1307–1313. 10.1007/s11947-011-0595-6

[B3] Astudillo-Ordóñez C. E. Rodríguez P. (2018). Physicochemical parameters of avocado Persea americana Mill. cv. Hass (Lauraceae) grown in Antioquia (Colombia) for export. Cienc. Tecnol. Agropec. 19, 383–392. 10.21930/rcta.vol19_num2_art:694

[B4] Bernal J.. (2016). Estudios ecofisiológicos en aguacate cv. Hass en diferentes ambientes como alternativa productiva en Colombia (Ph.D. thesis). Universidad Nacional de Colombia, Medellin, Colombia.

[B5] Bertling I. Bower J. P. (2006). Avocado sugars during early fruit development. South African Avocado Growers' Assoc. Yearb. 29, 38–39.31293606

[B6] Blakey R. J.. (2016). Evaluation of avocado fruit maturity with a portable near-infrared spectrometer. Postharvest Biol. Technol. 121, 101–105. 10.1016/j.postharvbio.2016.06.016

[B7] Carvalho C. P. Bernal E. J. Velásquez M. A. Cartagena V. J. R. (2015). Fatty acid content of avocados (Persea americana Mill. cv. Hass) in relation to orchard altitude and fruit maturity stage. Agron. Colomb. 33, 220–227. 10.15446/agron.colomb.v33n2.49902

[B8] Carvalho C. P. Velásquez M. A. Van Rooyen Z. (2014). Determination of the minimum dry matter index for the optimum harvest of “Hass” avocado fruits in Colombia. Agron. Colomb. 32, 399–406. 10.15446/agron.colomb.v32n3.46031

[B9] Dixon J. Cotterell C. Hofstee B. Elmsly T. A. (2009). University of California avocado cultivars “lamb hass” and “gem” maturity and fruit quality results from new zealand evaluation trials. New Zealand Avocado Growers' Assoc. Ann. Res. Rep. 8, 15–26.

[B10] Donetti M. Terry L. A. (2014). Biochemical markers defining growing area and ripening stage of imported avocado fruit cv. Hass. Journal of Food Composition and Analysis 34, 90–98. 10.1016/j.jfca.2013.11.011

[B11] Dorey E. Fournier P. Léchaudel M. Tixier P. (2016). A statistical model to predict titratable acidity of pineapple during fruit developing period responding to climatic variables. Sci. Hortic. 210, 19–24. 10.1016/j.scienta.2016.07.014

[B12] FAO. (2017). Avocado cultivation boom in South Lebanon. Available online at: https://www.weeportal-lb.org/news/avocadocultivation-boom-south-lebanon (accessed October 23, 2017).

[B13] Ferreyra E. Defilippi B. (2012). Factores de Precosecha que Afectan la Postcosecha de Palta Hass: Clima, Suelo y Manejo. INIA Bull. No. 248 (La Cruz, Chile: Centro Regional de Investigació n La Cruz, Instituto de Investigaciones Agropecuarias).

[B14] Hernández-Muñoz P. Almenar E. Ocio M. J. Gavara R. (2006). Effect of calcium dips and chitosan coatings on postharvest life of strawberries (Fragaria x ananassa). Postharvest Biol. Technol. 39, 247–253. 10.1016/j.postharvbio.2005.11.006

[B15] Hofman P. J. Jobin-Décor M. Giles J. (2000). Percentage of Dry Matter and Oil Content Are Not Reliable Indicators of Fruit Maturity or Quality in Late-harvested ‘Hass' Avocado. HortSci. 35, 694–695. 10.21273/HORTSCI.35.4.69422868147

[B16] Kader A. A.. (1999). “Fruit maturity, ripening, and quality relationships”, in International Symposium Effect of Pre-/& Postharvest Factors in Fruit Storage 485. p. 203–208. 10.17660/ActaHortic.1999.485.27

[B17] Kaiser C. Smith M. Wolstenholme B. (1992). Overview of lipids in the avocado fruit, with particular reference to Natal Midlands. South African Avocado Growers' Assoc. Yearb. 15, 78–82.

[B18] Kassim A. Workneh T. S. (2020). Influence of postharvest treatments and storage conditions on the quality of Hass avocados. Heliyon 6, e04234. 10.1016/j.heliyon.2020.e04234 32642570 PMC7334236

[B19] Kassim A. Workneh T. S. Bezuidenhout C. N. (2013). A review on postharvest handling of avocado fruit. Afr. J. Agr. Res. 19, 2385–2402. 10.5897/AJAR12.1248

[B20] Kokawa M. Hashimoto A. Li X. Tsuta M. Kitamura Y. (2020). Estimation of ‘Hass' Avocado (Persea americana Mill.) Ripeness by Fluorescence Fingerprint Measurement. Food Anal. Method. 13, 892–901. 10.1007/s12161-020-01705-7

[B21] Landahl S. Meyer M. D. Terry L. A. (2009). Spatial and temporal analysis of textural and biochemical changes of imported avocado cv. Hass during fruit ripening. J. Agric. Food Chem. 57, 7039–7047. 10.1021/jf803669x 19580285

[B22] Lee S.-K.. (1981). A review and background of the avocado maturity standard. Califor. Avoc. Soc. 65,101–109.

[B23] Lee S.-K. Young R. E. Schiffman P. M. Coggins C. W. J. (1983). Maturity studies of avocado fruit based on picking dates and dry weight. J. Amer. Soc. Hort. Sci. 108, 390–394.

[B24] Morton J.. (1987). “Avocado”, in Fruits of Warm Climates. (Miami: Florida Flair Books), p. 91–102.

[B25] Obenland D. Collin S. Sievert J. Negm F. Arpaia M. L. (2012). Influence of maturity and ripening on aroma volatiles and flavor in ‘Hass' avocado. Postharvest Biol. Technol. 71, 41–50. 10.1016/j.postharvbio.2012.03.006

[B26] Olarewaju O. O.. (2014). Evaluation of Maturity Parameters of ‘Fuerte' and ‘Hass' Avocado Fruit. 156. M. Sc. Agric. Durban: University of KwaZulu-Natal.

[B27] Osuna-García J. A. Doyon G. Salazar-García S. Goenaga R. González-Durán I. J. L. (2010). Effect of harvest date and ripening degree on quality and shelf life of Hass avocado in Mexico. Fruits 65, 367–375. 10.1051/fruits/2010031

[B28] Ozdemir F. Topuz A. (2004). Changes in dry matter, oil content and fatty acids composition of avocado during harvesting time and post-harvesting ripening period. Food Chem. 86, 79–83. 10.1016/j.foodchem.2003.08.012

[B29] Ranney C. A. Gillette G. Brydon A. McIntyre S. Rivers O. Vasquez C. A.. (1992). “Physiological maturity and percent dry matter of california avocado”, in Proceedings of Second World Avocado Congress. p. 379–385.

[B30] Rokaya P. R. Baral D. R. Gautam D. M. Shrestha A. K. Paudyal K. P. (2016). Effect of postharvest treatments on quality and shelf life of mandarin. AJPS 07, 1098–1105. 10.4236/ajps.2016.7710535432981

[B31] Snijder B. Mathumbu J. M. Kruger F. J. (2003). Development of fruit maturity and mineral content norms for export avocado cultivars from different south african avocado growing regions. South African Avocado Growers' Assoc. Yearb. 26, 51–54.

[B32] Taiti C. Costa C. Menesatti P. Caparrotta S. Bazihizina N. Azzarello E.. (2015). Use of volatile organic compounds and physicochemical parameters for monitoring the post-harvest ripening of imported tropical fruits. Eur. Food Res. Technol. 241, 91–102. 10.1007/s00217-015-2438-6

[B33] Vallejo-Pérez M. R. Téliz-Ortiz D. Colinas-León M. T. De La Torre-Almaraz R. Valdovinos-Ponce G. Nieto-Ángel D.. (2015). Alterations induced by Avocado sunblotch viroid in the postharvest physiology and quality of avocado ‘Hass' fruit. Phytoparasitica 43, 355–364. 10.1007/s12600-015-0469-y

[B34] Waissbluth R. Valenzuela J. (2007). Determination of the Minimum Percentage of Dry Matter to Authorize the Harvest of Hass Avocado Pears for Export. Paper presented at: VI World Avocado Congress (Viñ a Del Mar, Chile).

[B35] White A. Woolf A. B. Harker R. Davy M. (1999). Measuring avocado firmness: assessment of various methods. Rev. Chapingo Serie Hortic. 5, 389–392.

[B36] Xi W. Zheng H. Zhang Q. Li W. (2016). Profiling taste and aroma compound metabolism during apricot fruit development and ripening. Int. J. Mol. Sci. 17, 998. 10.3390/ijms17070998 27347931 PMC4964374

