# Supplementary material for: Evaluation of Postharvest Maturity Indices of Commercial Avocado Varieties Grown at Various Elevations Along Lebanon's Coast
Source: Front Plant Sci. 2022 Jun 15;13:895964. doi: 10.3389/fpls.2022.895964 (PMC9240439; doi:10.3389/fpls.2022.895964)
Supplement: Supplementary file 2 [file Presentation_2.pptx]

## Slide 1
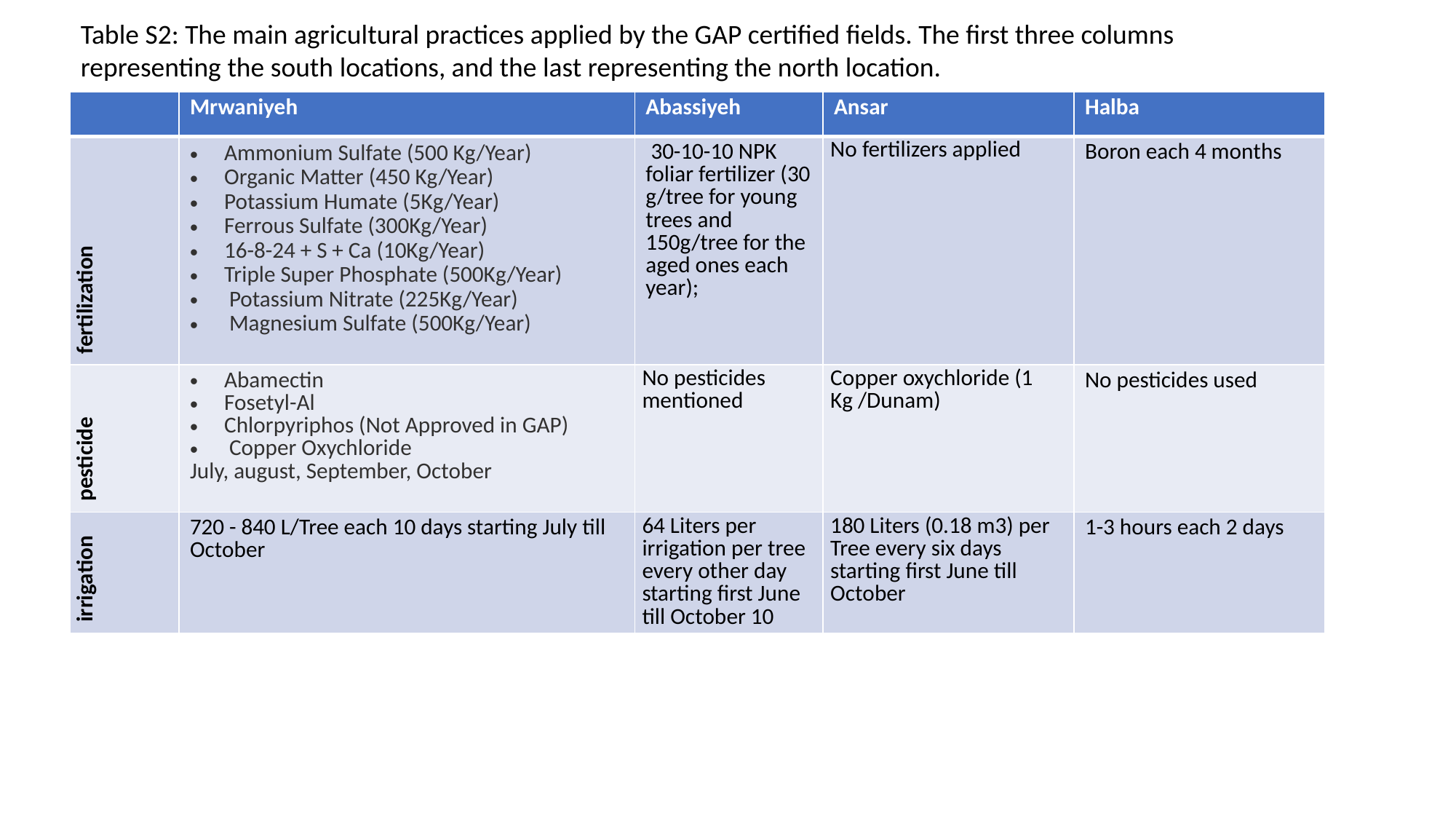

Table S2: The main agricultural practices applied by the GAP certified fields. The first three columns representing the south locations, and the last representing the north location.
| | Mrwaniyeh | Abassiyeh | Ansar | Halba |
| --- | --- | --- | --- | --- |
| fertilization | Ammonium Sulfate (500 Kg/Year) Organic Matter (450 Kg/Year) Potassium Humate (5Kg/Year) Ferrous Sulfate (300Kg/Year) 16-8-24 + S + Ca (10Kg/Year) Triple Super Phosphate (500Kg/Year)  Potassium Nitrate (225Kg/Year)  Magnesium Sulfate (500Kg/Year) | 30-10-10 NPK foliar fertilizer (30 g/tree for young trees and 150g/tree for the aged ones each year); | No fertilizers applied | Boron each 4 months |
| pesticide | Abamectin Fosetyl-Al Chlorpyriphos (Not Approved in GAP)  Copper Oxychloride July, august, September, October | No pesticides mentioned | Copper oxychloride (1 Kg /Dunam) | No pesticides used |
| irrigation | 720 - 840 L/Tree each 10 days starting July till October | 64 Liters per irrigation per tree every other day starting first June till October 10 | 180 Liters (0.18 m3) per Tree every six days starting first June till October | 1-3 hours each 2 days |
